# Synthesis of Nixantphos Core-Functionalized Amphiphilic Nanoreactors and Application to Rhodium-Catalyzed Aqueous Biphasic 1-Octene Hydroformylation

**DOI:** 10.3390/polym12051107

**Published:** 2020-05-12

**Authors:** Ahmad Joumaa, Florence Gayet, Eduardo J. Garcia-Suarez, Jonas Himmelstrup, Anders Riisager, Rinaldo Poli, Eric Manoury

**Affiliations:** 1 CNRS, LCC (Laboratoire de Chimie de Coordination), Université de Toulouse, UPS, INPT, 205 route de Narbonne, BP 44099, F-31077 Toulouse CEDEX 4, France; ahmad.joumaa@outlook.fr (A.J.); florence.gayet@lcc-toulouse.fr (F.G.); 2 Centre for Catalysis and Sustainable Chemistry, Department of Chemistry, Technical University of Denmark, Kemitorvet, Building 207, 2800 Kgs. Lyngby, Denmark; eduardoj.garcia@tecnalia.com (E.J.G.-S.); s144159@student.dtu.dk (J.H.); ar@kemi.dtu.dk (A.R.); 3 Institut Universitaire de France, 1 rue Descartes, 75231 Paris CEDEX 05, France

**Keywords:** aqueous biphasic catalysis, hydroformylation, polymerization-induced self-assembly, RAFT polymerization, rhodium, nixantphos, water-confined polymeric nanoreactors

## Abstract

A latex of amphiphilic star polymer particles, functionalized in the hydrophobic core with nixantphos and containing P(MAA-co-PEOMA) linear chains in the hydrophilic shell (nixantphos-functionalized core-crosslinked micelles, or nixantphos@CCM), has been prepared in a one-pot three-step convergent synthesis using reversible addition-fragmentation chain transfer (RAFT) polymerization in water. The synthesis involves polymerization-induced self-assembly (PISA) in the second step and chain crosslinking with di(ethylene glycol) dimethacrylate (DEGDMA) in the final step. The core consists of a functionalized polystyrene, obtained by incorporation of a new nixantphos-functionalized styrene monomer (nixantphos-styrene), which is limited to 1 mol%. The nixantphos-styrene monomer was synthesized in one step by nucleophilic substitution of the chloride of 4-chloromethylstyrene by deprotonated nixantphos in DMF at 60 °C, without interference of either phosphine attack or self-induced styrene polymerization. The polymer particles, after loading with the [Rh(acac)(CO)_2_] precatalyst to yield Rh-nixantphos@CCM, function as catalytic nanoreactors under aqueous biphasic conditions for the hydroformylation of 1-octene to yield *n*-nonanal selectively, with no significant amounts of the branched product 2-methyl-octanal.

## 1. Introduction

The hydroformylation of α-olefins, leading to a mixture of linear and branched aldehydes, is one of the most important homogeneously catalyzed reactions implemented in industry [1,2,3]. While rhodium affords better results in terms of activity and selectivity in favor of the desired linear products [4], the hydroformylation of higher olefins is still based on the less selective cobalt technology, because the much greater cost of the metal makes the process non-economical in the absence of an efficient catalyst recovery and recycle protocol [5,6]. Rhodium-catalyzed homogeneous processes are currently implemented only for the lighter olefins (i.e., propene, butene) because the aldehyde products are sufficiently volatile for recovery by distillation at relatively low temperatures, where the catalyst does not degrade [7]. An alternative biphasic approach, making use of water-soluble phosphines such as triphenylphosphine trisulfonate (TPPTS) to completely confine the rhodium catalyst in the aqueous phase, has been implemented successfully in industry since the mid 1980’s [8,9]. However, this methodology is also limited to the lightest α-olefins because of the need of sufficient water-solubility to afford reasonable mass transport rates toward the catalytic phase [10].

The efficient extension of aqueous biphasic catalysis to hydroformylation of higher α-olefins remains an outstanding challenge, despite several approaches having been proposed and studied. These approaches are surveyed in several recent reviews [11,12,13,14], and include the addition of modifiers or compatibilizers [15] such as cosolvents (mostly methanol, ethanol) [16,17,18,19,20,21,22], amphiphilic/thermo-regulated ligands and cyclodextrins [23,24,25] to improve mass transport as well as surfactants (cationic, anionic, double long chain cationic) to increase the interfacial area by emulsion and microemulsion methods [26,27,28,29,30,31,32,33,34,35,36,37]. Other interesting approaches apply thermomorphic methods with catalyst anchoring on lower critical solution temperature (LCST) polymers that become lipophilic at high temperatures [38,39,40,41,42,43,44,45] and on micellar formation with catalyst anchoring on the hydrophobic tail of surfactants [46,47,48]. Note that, in the literature, the term “micellar catalysts” is often used for the “mini-emulsion approach” with dramatic increase of the interface area by swollen micelles [49,50,51,52]. However, the catalyst is confined in the aqueous phase and the catalytic process occurs within the interface layer, whereas in what we are terming “micellar approach” the catalyst and the substrate are both located in the hydrophobic environment of the micellar nanoreactors.

**Scheme 1:**
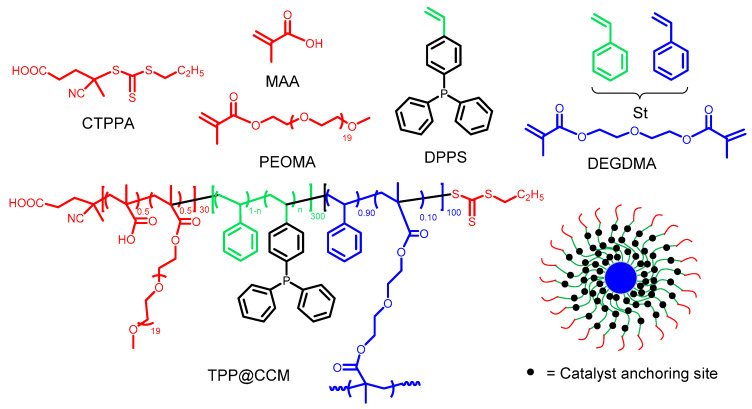
Monomers used for the synthesis of triphenylphosphine-containing core-cross-linked micelles, structure of the resulting polymer (TPP@CCM), and drawing of a schematic representation of the polymer architecture. MAA = methacrylic acid; PEOMA = poly(ethylene oxide) methyl ether methacrylate; St = styrene; DPPS = diphenylphosphinostyrene; DEGDMA = di(ethylene glycol) dimethacrylate.

An advanced version of the micellar approach for aqueous biphasic hydroformylation has recently been introduced in one of our groups [53,54,55,56,57,58]. It consists of replacing the ligand-functionalized self-assembled micelles with unimolecular equivalents named core-cross-linked micelles (CCMs), see Scheme 1. These objects were conveniently assembled in a one-pot three-step synthetic procedure by reversible addition-fragmentation chain transfer (RAFT) polymerization implemented in water, using the chain transfer agent 4-cyano-4-thiothiopropylsulfanyl pentanoic acid (CTPPA) and the monomers shown in Scheme 1 [53]. The catalyst anchoring site is a triphenylphosphine (TPP), which was installed within the hydrophobic nanoreactor core via the copolymerization of diphenylphosphinostyrene (DPPS) and regular styrene during the second step of the synthesis. All the micellar chains were cross-linked together at the core by di(ethylene glycol) dimethacrylate (DEGDMA) in the third step of the synthesis, to yield the final nanoreactors, named TPP@CCM. The molecular [Rh(acac)_2_(CO)_2_] precatalyst could be easily transported to the core of the nanoreactors where it coordinates to the phosphine ligands and yields [Rh(acac)(CO)(TPP)]@CCM [53]. The resulting objects were then used in aqueous biphasic hydroformylation of 1-octene to yield mostly linear (l) and branched (b) nonanals, with a typical l/b ratio of 4–5 (plus a minor amount of isomerization to internal octenes) [53,54,56,58]. The active hydride catalyst was generated in situ from [Rh(acac)(CO)(TPP)]@CCM in the presence of syngas under the hydroformylation conditions. High activities were obtained and the catalyst recovery was facile by room temperature decantation, with subsequent recycles showing only a minor activity decrease. Interestingly, a comparison between the core-crosslinked and the precursor non-crosslinked micelles (self-assembled from the synthetic intermediate prior to the final cross-linking step) showed only a slightly smaller activity [53], but at the same time a significantly smaller Rh leaching [57] for the CCM nanoreactor. Other studies also revealed that leaching is strictly related to the polymer transfer to the product phase and to Rh loss from the polymer [54].

**Scheme 2:**
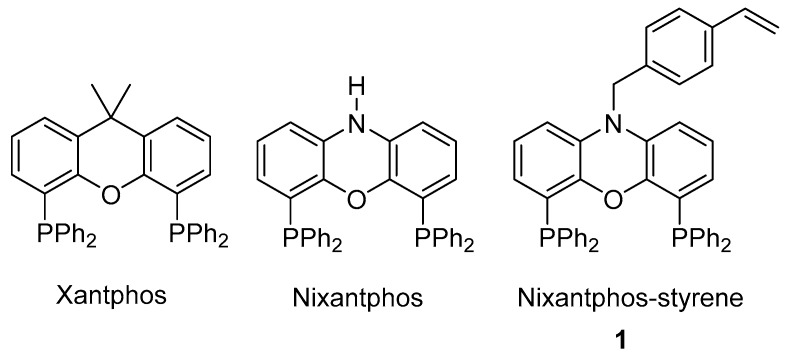
Large bite-angle chelating diphosphines that give high l/b regioselectivity in the Rh-catalyzed hydroformylation of α-olefins.

The synthetic modularity allows facile modification of the nanoreactor architecture, including the incorporation of different ligands. This requires the availability of a polymerizable version of the desired ligand. Indeed, an analogous nanoreactor incorporating a bis(4-methoxyphenyl)phenylphosphine, BMOPPP@CCM, has already been synthesized and tested in 1-octene hydroformylation [55]. Bidentate ligands, especially those with a large bite angle like xantphos [59], have been shown to yield higher l/b ratios, which is of primary interest for practical applications. It was therefore decided to develop a modified version of the above described functionalized CCM, incorporating a suitable chelating ligand. For practical reasons related to the functionalized monomer synthesis, the 4,5-bis(diphenylphosphino)phenoxazine (nixantphos) ligand [60] was selected. This is an analogue of xantphos [61] where N-H replaces the CMe_2_ group (see Scheme 2). The N-H function imparts suitable reactivity to the molecule for ligand anchoring on various substrates. It has already been exploited for direct immobilization on isocyanate-functionalized polystyrene [62] and for the installment of other suitable functions leading to a covalent [63] or non-covalent [64] immobilization on silica or alumina, or on dendritic supports [65]. Therefore, we have considered it suitable for the introduction of a polymerizable styrene unit. We report here the synthesis of the new functionalized monomer, N-(4-styrylmethyl)-4,5-bis(diphenylphosphino)phenoxazine that we shall abbreviate as nixantphos-styrene (**1**, Scheme 2), the synthesis and characterization of the nixantphos@CCM nanoreactors, and preliminary studies of their performance in the aqueous biphasic hydroformylation of 1-octene.

## 2. Materials and Methods

### 2.1. Materials

Unless otherwise stated, all operations were carried out under an atmosphere of prepurified argon using standard Schlenk line techniques. The solvents were dehydrated by conventional methods and distilled under argon or thoroughly degassed prior to use. Compounds 4,5-bis(diphenylphosphino)phenoxazine (nixantphos) (98%, Strem Chemicals, Newburyport, MA, US), *p*-chloromethylstyrene (90% techn., Acros Organics, Geel, Belgium), 4,4′-azobis(4-cyanopentanoic acid) (ACPA, >98%, Fluka), methacrylic acid (MAA, 99.5%, Acros Organics), poly(ethylene oxide) methyl ether methacrylate (PEOMA, with an average of 20 ethylene oxide units, M_n_ = 950 g moL^-1^, Sigma-Aldrich, St. Louis, MO, US), di(ethylene glycol) dimethacrylate (DEGDMA, 95%, Sigma-Aldrich), 1,3,5-trioxane (>99%, Sigma-Aldrich), [Rh(acac)(CO)_2_] (99%, Alfa Aesar, Haverhill, MA, US), 1-octene (98%, Sigma-Aldrich), *n*-nonanal (95%, Sigma-Aldrich), *n*-decanal (≥98%, Sigma-Aldrich), *n*-dodecane (≥99%, Sigma-Aldrich) and decane (≥99%, Sigma-Aldrich) as external standard were used as received unless otherwise stated. Styrene (99%, Acros Organics) was purified through a column of active basic aluminum oxide to remove the stabilizer. The RAFT chain transfer agent 4-cyano-4-thiothiopropylsulfanyl pentanoic acid (CTPPA) was synthesized as described previously [66,67]. The hydroformylation reaction gases CO (99.97%), H_2_ (99.999%) and N_2_ (99.99%) were obtained from Air Liquide, Denmark and used as received. A customized stainless steel high-pressure autoclave (25 mL volume) equipped with manometer, thermoelement and pressure relief valve was used for the hydroformylation reactions with heating provided by a temperature-controlled aluminium block positioned on a magnetic stirrer.

### 2.2. Instrumentation

NMR: ^1^H NMR and ^31^P NMR measurements were performed in 5 mm diameter tubes in DMSO-*d*_6_ or THF-*d*_8_ solution at 25 °C using a Bruker Avance 300 spectrometer. The chemical shift scale was calibrated on the basis of the solvent peak (δ = 2.50 ppm for DMSO; δ = 3.58 and 1.73 ppm for THF). For the polymerization monitoring, the monomer consumption was measured by adding aliquots of the polymerization medium directly dissolved in the NMR solvent in the presence of 1,3,5-trioxane as an integration reference (δ = 5.20 ppm). For the CCM characterization, 1 mL of latex was dried under vacuum until a white paste was obtained, then the latter was dispersed in the NMR solvent.

SEC: The size exclusion chromatographic analyses were carried out using a flow rate of 1 mL/min on a system equipped in series with a Wyatt Technology Optilab rEX differential refractometer, a Wyatt technology MiniDAWN multiangle static light scattering detector (MALS) and a VarianProstar UV-Vis spectrometer. The samples were dried under vacuum and then dissolved in anhydrous THF at concentrations of 5 mg/mL.

DLS: The intensity-average diameters of the latex particles (Dz) and the polydispersity index (PDI) were measured at 25 °C on a Malvern Zetasizer NanoZS. After filtration through a 0.45 µm pore-size membrane of regenerated cellulose, deionized water or THF was used to dilute the latex sample. Solutions were analyzed without further filtration to ensure that undesired populations were not removed. Data were analyzed by the general-purpose non-negative least squares (NNLS) method. The typical accuracy for these measurements was 10%–15%.

TEM: Diluted latex samples were dropped on a formvar/carbon-coated copper grid and dried in air. The samples were examined with a JEM-1100 transmission electron microscope equipped with a tungsten filament operating at 100 kV (Centre de MicroCaractérisation Raimond Castaing of the University of Toulouse).

GC-MS: Conversion and product selectivity after hydroformylation reactions were determined by GC-MS analysis of a 30 μL aliquot of the organic reaction phase diluted with 850 μL of ethyl acetate and 120 μL of external standard. The GC-MS instrument was an Agilent 6850 GC system with a Varian CP7502 capillary column including an Agilent 5975C VL MSD with triple-axis detector with helium as carrier gas.

### 2.3. Synthesis of the Ligand-Functionalized Styrene Monomer

Synthesis of N-(4-styrylmethyl)-4,5-bis(diphenylphosphino)phenoxazine (**1**): To a solution of bis(diphenylphosphino)phenoxazine (0.500 g, 0.907 mmol) in dimethylformamide (5 mL) was added a solution of NaH (44 mg, 1.83 mmol) in dimethylformamide (5 mL). The resulting mixture was heated at 70 °C for 1.5 h. After cooling to room temperature, 4-chloromethylstyrene (277 mg, 1.82 mmol) was added, followed by heating at 60 °C for 18 h. After filtration, the solution was evaporated to dryness under reduced pressure and the solid residue was washed by degassed cyclohexane and dried under vacuum. The crude product was purified by column chromatography on silica gel, eluting with dichloromethane, yielding the pure product as a white solid (460 mg, 76%). ^1^H NMR (CDCl_3_): δ 7.42 (2H, d, *J* = 8.2 Hz, styryl); δ 7.29 (2H, d, *J* = 8.2 Hz, styryl); 7.27–7.20 (20H, m, PPh_2_); 6.73 (1H, dd, *J* = 17.6, 10.9 Hz, C*H*=CH_2_); 6.59 (2H, br t, *J* = 7.8 Hz, xanthene CHC*H*CH); 6.31 (2H, br d, *J* = 7.8 Hz; xanthene C*H*CHCH); 6.05 (2H, br d, *J* = 7.8 Hz, xanthene CHCHC*H*); 5.76 (1H, br d, *J* = 17.6 Hz: C*H*_2_=CH); 5.26 (1H, br d, *J* = 10.9 Hz, C*H*_2_=CH); 4.80 (2H, s, N-C*H*_2_). ^31^P NMR (CDCl_3_): δ −19.0. ^13^C NMR (CDCl_3_): δ 147.42 (virtual t, *J* = 20.9 Hz, xanthene *C*-P or *C*-O); 136.86 (virtual t, *J* = 12.3 Hz, *i-*PPh_2_); 136.70 (*i-*styryl); 136.30 (vinyl CH); 135.63 (*i*’-styryl); 133.88 (virtual t, *J* = 20.8 Hz, *o*-PPh_2_); 133.71 (virtual t, *J* = 24.0 Hz, xanthene *C*-N); 128.24 (*p*-PPh_2_); 128.14 (vitual t, *J* = 6.9 Hz, *m*-PPh_2_); 126.78 (styryl CH); 126.29 (styryl CH); 125.62 (xanthene CH); 124.76 (virtual t, *J* = 19.1 Hz, xanthene *C*-O or *C*-P); 123.76 (xanthene CH); 113.91 (vinyl CH_2_); 112.76 (xanthene CH); 49.72 (N-*C*H_2_). NB: For the virtual triplets, the J_PC_ values are determined as the chemical shift difference between the two external resonances.

Phosphorus protection in 4,5-bis(diphenylphosphino)phenoxazine. Synthesis of 4,5-bis(thiodiphenylphosphino)phenoxazine (**2**): 4,5-bis(diphenylphosphino)phenoxazine (0.100 g, 0.181 mmol) and elemental sulfur (29 mg, 0.9 mmol) were dissolved in degassed 1,2-dichloroethane (2 mL) and the resulting solution was heated at 83 °C overnight. After filtration, the solution was evaporated and the residue was purified by column chromatography on silica gel, eluting first with pentane and then with dichloromethane. The product was recovered as a white solid (100 mg, 90%). ^1^H NMR (CDCl_3_): δ 7.66–7.38 (20H, m, PPh_2_); 6.48 (2H, t, *J* = 6.5 Hz, xanthene CHC*H*CH); 6.37 (2H, d, *J* = 7.6 Hz, xanthene C*H*CHCH); 5.86 (2H, m, xanthene CHCHC*H*); 2.29 (1H, s, N*H*). ^31^P NMR (CDCl_3_): δ 41.5.

### 2.4. Synthesis of the Nixantphos@CCM Latex

This procedure closely follows the one previously reported for the preparation of TPP@CCM [53].

First step: R_0_-(MAA_0.5_-co-PEOMA_0.5_)_28_-SC(S)SPr (macroRAFT-1): In a Schenk tube was prepared a stock solution of ACPA (100 mg) and NaHCO_3_ (100 mg) in 1 mL of deionized water. 400 μL of this solution (40 mg of ACPA, 0.143 mmol), CTPPA (211 mg, 0.762 mmol), MAA (953 mg, 11.1 mmol), PEOMA (10.36 g, 10.9 mmol) and 44.1 g of deionized water were introduced in the reaction flask. 1,3,5-Trioxane was added to the mixture as internal reference for the determination of the monomer conversion by ^1^H NMR. The total mass and total volume were 55.7 g and 54.8 mL. The flask was closed with a rubber septum and then purged with an argon flow for 45 min. It was then heated with magnetic stirring for 2 h at 80 °C. The monomer conversion (^1^H NMR in DMSO-*d*_6_) was 98%, yielding a theoretical polymer mass of 11.3 g (M_n,th_ = 14,800 g mol^−1^). M_n_ (SEC in THF) = 12100 g moL^−1^; Ð = 1.18.

Second step: R_0_-(MAA_0.5_-co-PEOMA_0.5_)_28_-b-St_56_-SC(S)SPr (macroRAFT-2): A portion of the solution obtained from the first step (5.3 mL, 1.096 g of macroRAFT agent, 0.074 mmol), after dilution with 0.7 mL of deionized water and degassing with an argon purge, was treated with styrene (432 mg, 4.15 mmol, 56.0 equiv. per macroRAFT) and with a portion of the ACPA stock solution (41 µL, 4.1 mg of ACPA, 0.015 mmol). The total mass and total volume were 6.53 g and 6.52 mL. The flask was heated for 3 h at 80 °C, yielding complete conversion and a white translucent final suspension. Theoretical mass = 1.528 g; M_n,th_ = 20,600 g mol^−1^.

Third step: R_0_-(MAA_0.5_-co-PEOMA_0.5_)_28_-b-St_56_-b-(St_0.991_-co-**1**_0.009_)_274_-SC(S)SPr (nixantphos@M): The suspension resulting from the second step was diluted with 7 g of deionized water and degassed by an argon purge, while a solution of nixantphos (122.0 mg, 0.183 mmol, 2.47 equiv. per macroRAFT agent) in styrene (2.10 g, 20.15 mmol, 271.9 equiv. per macroRAFT agent) was prepared in a separate flask. The two mixtures were combined and a portion of the ACPA stock solution (41 µL, 4.1 mg of ACPA, 0.015 mmol) was added. The total mass and total volume were 15.79 g and 15.88 mL. The mixture was heated for 3 h at 80 °C, yielding complete conversion and a white opaque colloidal suspension. Theoretical mass = 3.75 g; M_n,th_ = 50,600 g mol^−1^.

Fourth step: Core cross-linking to produce R_0_-(MAA_0.5_-co-PEOMA_0.5_)_28_-b-St_56_-b-(St_0.991_-co-**1**_0.009_)_274_-b-(St_0.89_-co-DEGDMA_0.11_)_105_-SC(S)SPr (nixantphos@CCM): The suspension resulting from the third step was further diluted with 10 g of deionized water and degassed by an argon purge, while a mixture of DEGDMA (220 mg, 0.908 mmol, 12.3 equiv. per macroRAFT agent) and styrene (730 mg, 7.00 mmol, 94.5 equiv. per macroRAFT agent) was prepared in a separate flask. The two mixtures were combined and a portion of the ACPA stock solution (50 µL, 5.0 mg of ACPA, 0.018 mmol) was added. The total mass and total volume were 26.75 g and 26.94 mL. The mixture was heated for 1.5 h at 80 °C, leading to a 98% monomer conversion. DLS of the final polymer in water: D_z_ = 33 nm; PDI = 0.26.

### 2.5. Hydroformylation Catalysis

Rh-nixantphos@CCM catalyst preparation: Catalyst solutions were prepared by transferring nixantphos@CCM (0.225 mL) and deionized, degassed water (1.6 mL) into a 10 mL Schlenk flask followed by stirring for 15 min. Then organic solvent (0.15 mL) was added followed by stirring for another 5 min, before Rh precursor solution (0.150 mL containing 1.1 mg of [Rh(acac)(CO)_2_] per mL of solvent) were added and the mixture stirred for additionally 5 min. The fresh catalyst solution was stored under inert gas until transferred to the high-pressure reactor for performing the hydroformylation experiments.

Hydroformylation of 1-octene: The hydroformylation reactions were performed in a 25 mL high-pressure batch reactor. The reactor was initially purged with N_2_ gas where after a desired amount of the prepared Rh-nixantphos@CCM catalyst solution (see above), organic solvent (2.45 mL) and 1-octene (0.565 mL) were transferred to the reactor consecutively by cannula. The reactor was then purged with syngas mixture (1:1 mixture of CO and H_2_), pressurized to a set-pressure and heated to a selected set-temperature, where after the reaction was performed for a desired reaction time under magnetic stirring (800 rpm). After reaction, the reactor was cooled to room temperature, depressurized and the reaction mixture transferred to a separation funnel in order to separate the aqueous phase containing the Rh-catalysts and the organic phase containing the products. Conversions and reaction selectivities were calculated by means of GC-MS analysis of aliquots of the organic phase.

Catalyst recycling tests: The separated aqueous phase containing the Rh-nixantphos@CCM catalyst was extracted (3 × 1.5 mL) with the organic solvent employed as reaction media, and placed in the high-pressure reactor together with additional organic solvent (2.6 mL) and 1-octene (0.565 mL). Here after the same catalytic procedure as described above was followed.

## 3. Results and Discussion

### 3.1. Synthesis of the Ligand-Functionalized Monomer, ***1***

**Scheme 3:**
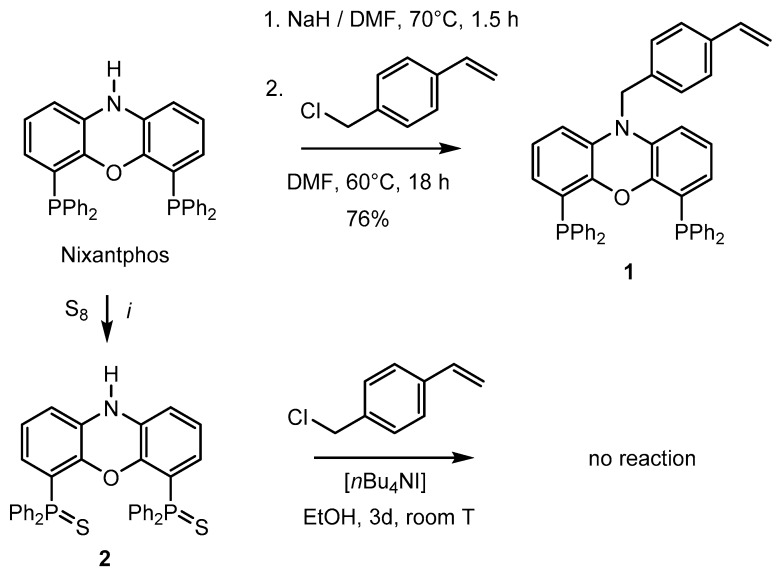
Synthesis of the nixantphos-styrene monomer.

The synthesis of the target ligand is in principle not straightforward. The obvious strategy is a nucleophilic substitution at the benzylic carbon of 4-chloromethylstyrene by the nixantphos N-H function. However, there are two potential competing processes: A self-initiated polymerization, particularly because the nucleophilic substitution reaction requires high temperature conditions, and the possible nucleophilic attack by the phosphorous atom. Initially, in order to avoid the latter phenomenon, we have proceeded to protect the phosphine functions by conversion to thiophosphines with elemental sulfur. This procedure has the added advantage of rendering the system air stable (nixantphos is a quite air sensitive molecule), thereby simplifying the compound purification procedure and handling operations. The oxidation reaction was actually less facile than expected with a reflux in dichloromethane for 24 h resulting only in partial conversion, but could eventually be rendered quantitative by heating overnight in dichloroethane at 83 °C to obtain the desired sulfur-protected nixantphos **2** (Scheme 3). However, all subsequent attempts to couple **2** and 4-chloromethylstyrene have not afforded the desired alkylation product. Even in the presence of *n*Bu_4_NI, which generates the more reactive 4-iodomethylstyrene in situ, there was no conversion after 3 days in ethanol at room temperature. It is likely that the electronic delocalization of the nixantphos nitrogen-lone pair into the xanthene skeleton makes the N-H function insufficiently nucleophilic to attack the benzylic CH_2_Cl function.

**Figure 1:**
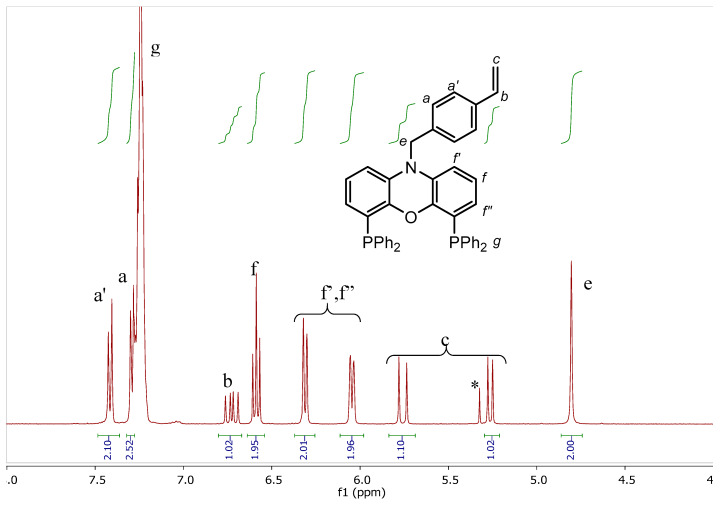
^1^H NMR of compound **1** in CDCl_3_. The starred resonance belongs to residual CH_2_Cl_2_.

We have then proceeded to exalt the N-atom nucleophilicity by deprotonation. In that case, the phosphine protection becomes superfluous because the anionic amide is a better nucleophile than the neutral phosphine. Indeed, the target monomer could be synthesized in 76% isolated yields by deprotonation with NaH in DMF and subsequent reaction with 4-chloromethylstyrene at 60 °C for 18 h. The excess NaH could be eliminated by filtration, while the excess 4-chloromethylstyrene was removed by washings with degassed cyclohexane. No polymerization or competing attack by the phosphine groups could be evidenced. The product was characterized by ^31^P, ^1^H and ^13^C NMR in CDCl_3_ and the assignments were aided by COSY, ^13^C{^1^H,^31^P}, ^1^H-^13^C{^31^P} HMQC and ^1^H-^13^C{^31^P} HMBC spectra (see Supporting Information, Figures S1–S5). The ^31^P NMR spectrum shows the free phosphine resonance at −19.0 ppm and the absence of any other resonance, attributable for instance to phosphorus oxidation (Figure S1). The ^1^H NMR spectrum (Figure 1) clearly shows the styryl aromatic protons as mutually coupled doublets at 7.42 and 7.29 ppm, as confirmed by the COSY spectrum (Figure S2). The PPh_2_ aromatic resonances are clustered in the 7.27–7.20 ppm region and the xanthene skeleton protons as observed as a set of three resonances (see COSY in Figure S2): a triplet at 6.59 ppm for the central proton (f) and two doublets at 6.31 and 6.05 ppm for the lateral ones (f’ and f”, ^3^J_HH_ = 7.8 Hz). Finally, the NCH_2_ protons (e) yield a singlet at 4.8 ppm. The characteristic resonances of the vinyl function are also visible, though the geminal coupling between the signals at 5.76 and 5.26 ppm is too weak and thus these signals are observed as doublets (*J* = 17.6 and 10.9 Hz, respectively), whereas the =CH- proton (b) is visible as a doublet of doublets at 6.73 ppm.

**Figure 2:**
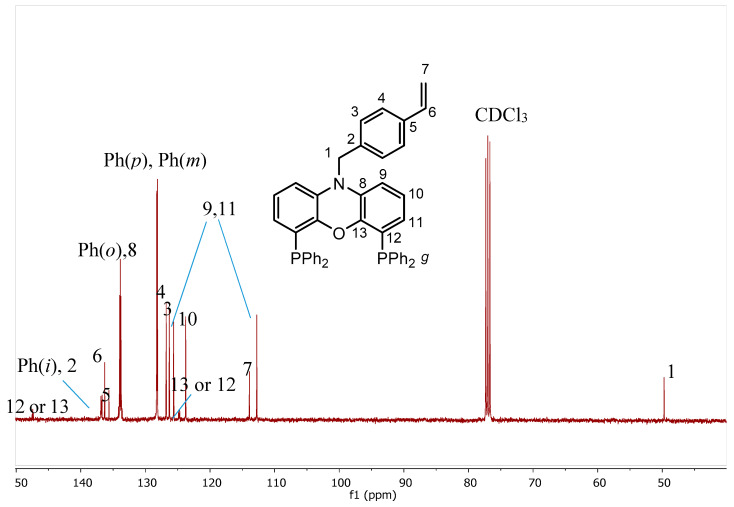
^13^C{^1^H} NMR of compound **1** in CDCl_3_.

The ^13^C{^1^H} spectrum is shown in Figure 2. All H-bearing carbon atoms (the vinyl atoms 6 and 7, the styryl atoms 1, 3 and 4, and the nixantphos atoms 9, 10 and 11) could clearly be assigned on the basis of the HMQC spectrum (Figure S4). The H atoms a and a’ can be differentiated because only a shows a long-range correlation with the carbon atom 1 in the HMBC spectrum (Figure S5). On this basis, we can attribute the styryl ipso C atoms 2 and 5 because atom 2 is correlated only to a and atom 5 only to a’. In addition, atom 5 also exhibits long-range correlations with protons b and c. The distinction between atoms 3 and 4 is based on the long-range correlations exhibited in the HMBC spectrum with the H atoms e for atom 3 and b for atom 4. Conversely, the H atoms f’ and f” are not unambiguously distinguishable from the HMBC long-range correlations. Atoms 9, 10 and 11 do not show visible coupling to the P nucleus. The P-Ph C atoms were easily assigned on the basis of their relatively high intensity and characteristic chemical shifts and J_CP_ (ipso: δ 136.86, J_CP_ = 12.3 Hz; ortho: δ 133.88, J_CP_ = 20.8 Hz; meta: δ 128.14, J_CP_ = 6.9 Hz; para: δ 128.25, J_CP_ = 0) in comparison with the literature [68]. Interestingly, all the P-coupled resonances are observed as virtual triplets because of a strong P-P coupling (cf. the ^13^C{^1^H} and the ^13^C{^1^H,^31^P} patterns of the relevant expanded regions in Figure S3). The three residual unsubstituted C atoms (labelled 8, 12 and 13) all show couplings as virtual triplets to the P atom at δ 147.42 (J_CP_ = 20.9 Hz), δ 133.71 (J_CP_ = 24.0 Hz) and δ 124.76 (J_CP_ = 19.1 Hz). Atom 8 is attributed on the basis of the long-range correlation with the H atom e, where atoms 12 and 13 cannot be clearly distinguished.

### 3.2. Synthesis and Characterization of the Nanoreactors Nixantphos@CCM

**Scheme 4:**
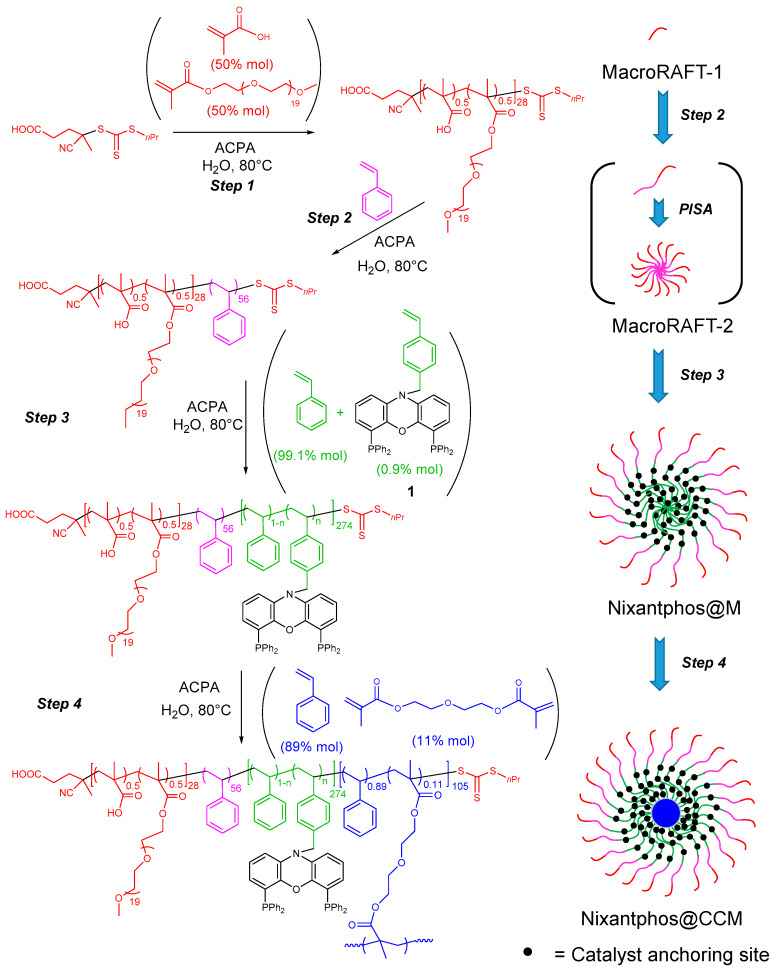
Synthesis of nixantphos@CCM.

The polymer synthesis made use of the RAFT method [69,70,71,72,73], which is a powerful technique based on degenerative radical exchange to implement what is known as “reversible deactivation radical polymerization” (RDRP) [74]. In addition, carrying out the synthesis in water allows implementation of the “polymerization-induced self-assembly” (PISA) principle [75,76,77]. The synthesis followed rather closely those previously reported for TPP@CCM and BMOPPP@CCM, except for the differences pointed out below. In the first step (see Scheme 4), the water-soluble chain transfer agent CTPPA (R_0_SC(S)SPr), in the presence of ACPA as an initiator and a 1:1 mixture of MAA and PEOMA (15 equivalents of each monomer, 30 in total, per CTPPA), yielded the product macroRAFT-1, consisting of water-soluble chains of average formula R_0_-(MAA_0.5_-*co*-PEOMA_0.5_)_28_-SC(S)SPr. In the previously reported TPP@CCM and BMOPPP@CCM syntheses [53,55], this macroRAFT intermediate was directly chain-extended with the mixture of styrene and the ligand-functionalized styrene monomer. Attempts to do the same in this case, however, even at the low 1% concentrations of monomer **1** in styrene that eventually led to the successful synthesis, induced the precipitation of **1** and the formation of an ill-defined product. This is presumably the consequence of the limited solubility of **1** in styrene and of the preferential incorporation of the latter monomer in the initial phases of the chain extension. To alleviate this problem, the synthetic procedure incorporated an additional step consisting of a chain extension with a short polystyrene block (56 monomer units on average, step 2 of Scheme 4) to yield the macroRAFT-2 intermediate, where the amphiphilic diblock copolymer chains have the average composition R_0_-(MAA_0.5_-co-PEOMA_0.5_)_28_-b-St_56_-SC(S)SPr. The hydrophobic block length in this intermediate is sufficient to induce PISA and generate micelles.

In step 3, copolymerization of styrene and **1** (99.1:0.9 molar ratio) eventually led to successful chain extension and generation of the ligand functionalized micelles, nixantphos@M, with the average composition R_0_-(MAA_0.5_-co-PEOMA_0.5_)_28_-b-St_56_-b-(St_0.991_-**1**_0.009_)_274_-SC(S)SPr for the individual chains. As already mentioned above, the success of this step (full incorporation of **1**) is limited by the solubility of **1** in the styrene monomer. Indeed, the procedure requires that all co-monomers are located in the same liquid phase, compatible with the growing hydrophobic micellar core. In the previous synthesis of TPP@CCM, it was possible to incorporate the ligand-functionalized monomer (DPPS) up to 25 mol% relative to styrene within the hydrophobic block [78], whereas the incorporation of bis(4-methoxyphenyl)phosphinostyrene was limited to 5 mol% [55]. Finally, step 4 was carried out, as in the previously reported syntheses [53,55], with DEGDMA as a cross-linker, used in combination with styrene (DEGDMA:St = 11:89), to yield the final product nixantphos@CCM. The large amount of styrene in this step is necessary to avoid macrogelation as already detailed in our previous work [53].

**Figure 3:**
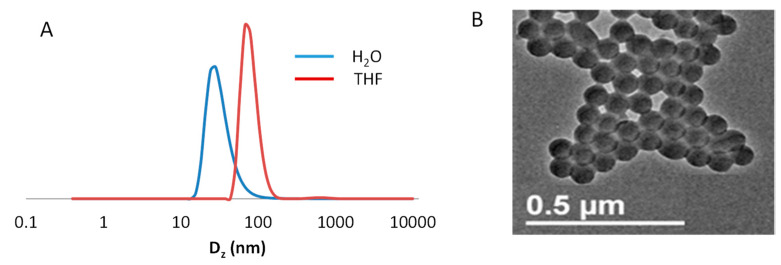
Characterization of the nixantphos@CCM particle size and morphology: DLS of dispersions in water and THF (**A**) and TEM image (**B**).

The DLS and TEM characterization confirmed that nixantphos@CCM consists of individual particles of spherical morphology and narrow size polydispersity, see Figure 3. Notably, the DLS measurement of the pristine latex shows a narrow size distribution with average diameter D_z_ = 33 nm (PDI = 0.26), whereas the dried polymer redispersed in THF shows D_z_ = 68 nm (PDI = 0.20), indicating a large swelling capacity for these particles. The size difference is related to the core swelling by THF. The average volume increases by a factor of 8.8, which compares with factors of 10.9 for TPP@CCM (D_z_ increase from 79 to 175 nm) [53] and 17.7 for BMOPPP@CCM (D_z_ increase from 81 to 207 nm) [55] for the same swelling procedure. For the previously reported TPP@CCM and BMOPPP@CCM nanoreactors, the controlled nature of the polymerization of both the hydrophilic and hydrophobic monomers and the efficiency of the reactivity switching between the two blocks was investigated in detail [53,55]. The change of functionalized comonomer to nixantphos-styrene in the polystyrene block, especially at the small 1% level, is not expected to negatively affect the polymerization controllability, which is confirmed by the size and size dispersity of the final product as shown in Figure 3.

**Figure 4:**
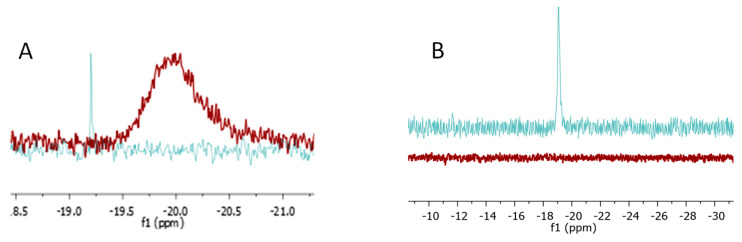
^31^P NMR spectra of nixantphos@CCM. (**A**) In THF-*d*_8_ (brown trace), with the spectrum of the monomer **1** also shows in light blue for comparison; (**B**) in D_2_O before (brown trace) and after (blue trace) swelling with toluene.

The ^31^P NMR spectrum confirms the presence of nixantphos (broad resonance at δ −20.0 in THF-*d*_8_, or at δ −19.0 in D_2_O after swelling with toluene; cf. sharp resonance at δ −19.24 for the monomer), see Figure 4. Most notably, the ^31^P NMR spectrum also indicates that the nixantphos ligand was not oxidized during the synthetic procedure (absence of a low-field resonance in the typical phosphine oxide region).

The ^1^H NMR characterization also provides information on the swelling process. In D_2_O, only the hydrophilic shell (PEO protons of the PEOMA monomer) is visible. The resonances of the protons in the unsolvated core are too broad because of the long correlation time (slow tumbling in the medium) and are not observed, like the ^31^P resonance of nixantphos. The core signals become, on the other hand, observable by either recording the spectrum in THF-*d*_8_ (Figure S8) or in D_2_O after core swelling by a good solvent for polystyrene, such as toluene (Figure S9) or dichloromethane, as previously shown for similar polymers incorporating other ligands [53,55,78]. The nixantphos ligand is not present in sufficient quantities to observe its distinct resonances in the ^1^H NMR spectrum, its resonances being masked by those of the dominant styrene units. Measurement of the toluene-swollen and equilibrated sample in D_2_O allows estimation of the toluene amount located inside the hydrophobic core by integration of the toluene resonances relative to those of the PEO protons. This gives a range of 730–810 toluene molecules per chain, depending on whether the calculation is based on the methyl or aromatic proton integration. The large uncertainty is caused by overlap of the toluene and polystyrene resonances. This range is similar to those found previously for the other related particles. The PEO resonances in the ^1^H NMR spectrum of the toluene-swollen sample in D_2_O reveal a core-shell interface structuring. The same phenomenon was previously observed and analyzed in detail for TPP@CCM [78], with a partitioning of the PEO chains between the water phase and the particle core (see the spectral deconvolution in Figure S10 and related discussion in the SI).

### 3.3. Biphasic Hydroformylation of 1-Octene

The influence of various reaction parameters, i.e., solvent, metal-loading, reaction time and pressure was examined for 1-octene hydroformylation using the prepared Rh-nixantphos@CCM catalyst and the results are compiled in Table 1. A preliminary temperature screening confirmed 90 °C to be a suitable reaction temperature, whereas 120 °C resulted in decomposition of the catalyst. Noteworthy, in all reactions essentially only the linear aldehyde *n*-nonanal, and not the branched product 2-methyl-octanal, was observed as hydroformylation product, confirming a very high regioselectivity of the catalyst to the linear product (estimated to ≥98.5%, i.e., l:b ratio ≥65). This regioselectivity was nearly the same as the 98.6% *n*-nonanal (l:b ratio 69) obtained for the homogeneous 1-octene hydroformylation in toluene with the Rh-nixantphos catalyst under similar reaction conditions (80 °C, 20 bar 1:1 CO/H_2_, P/Rh ratio 5) [60]. Moreover, it was slightly higher than for the reaction with 1-octene (neat or dissolved in CH_2_Cl_2_) using silica- or dentritic polyglycerol polymer-supported Rh-nixantphos catalysts, where l:b ratios of typically 30 (depending on the immobilization procedure and the support linkage spacer) were obtained even with a higher amount of ligand (P/Rh ratio 10) promoting the *n*-aldehyde formation [63,64,65].

Decanal, heptanal and dodecane were examined as potential organic solvents that would make a biphasic system after reaction, thus allowing facile separation of the Rh-nixantphos@CCM catalyst from the reaction products as well as re-use. Other organic solvents such as, e.g., toluene, 1-octanol and ethyl acetate proved unsuited for this purpose. As shown in Table 1, the highest yield of *n*-nonanal (7%) was obtained after 3 h of reaction with 0.18 mol% Rh catalyst loading at 90 °C and 20 bar pressure using decanal as solvent (entry 1). With heptanal as solvent, an aldehyde yield of 5% was obtained (entry 2), whereas no 1-octene conversion and aldehyde formation was observed in dodecane (entry 3) under similar reaction conditions. After reaction both the decanal and heptanal phases were slightly yellow colored, presumably due to Rh-catalyst leaching into the organic phase, and a relatively large amount of isomerization products were observed (mostly 2-octene and 3-octene). Furthermore, the phase separation was not as distinctive as in the reaction with dodecane, due to the partial catalyst solubility in both the organic and the aqueous phase. On the other hand, with dodecane as solvent (even though no conversion occurred) the organic phase remained colorless, the separation of the two phases was more facile than the other two tested solvents and no isomerization products formed.

The influence of increased Rh catalyst loading (0.36 mol%) at 90 °C and 20 bar total pressure was initially examined with decanal as organic solvent and resulted—as expected—in a higher yield of 12% of *n*-nonanal (entry 4). However, the use of this solvent remained hampered by Rh leaching, difficult phase separation and formation of 1-octene isomerization products. In contrast, *n*-nonanal formed in nearly the same yield (13%) at 90 °C and 20 bar total pressure with dodecane as solvent with no detectable isomerization products with the increased Rh catalyst loading at prolonged reaction time of 20 h (entry 5), thus clearly making dodecane the preferred solvent. Further prolongation of the reaction time from 20 to 72 h at 20 bar pressure did not increase the *n*-nonanal yield (14%), but resulted instead in significantly increased formation of isomerization products (entry 6). Oppositely, increase of the reaction pressure from 20 to 30 bar with a reaction time of 20 h improved the yield of *n*-nonanal to 28% (entry 7) without promoting formation of the undesired isomerization products, thus making these conditions optimal towards aldehyde formation.

As mentioned in the introduction, a detailed comparison of activity and Rh leaching in 1-octene hydroformylation was carried out for the related TPP@CCM nanoreactors, which have an identical polymer structure except for the substitution of TPP with nixantphos, and the corresponding non-crosslinked micelles. It was also established that both activity and leaching are related to the polymer architecture (mass transport, high-temperature lipophilicity) and not to the Rh coordination [53,54,57]. Furthermore, the nanoreactors recyclability and their stability in terms of size and polydispersity upon prolonged heating under the catalysis conditions were also detailed in those studies. These characteristics are not expected to change upon substitution of the core ligand and these tests were therefore not repeated for the current nanoreactor. However, an attempt of recovery and reuse of the catalyst was done in a recycling experiment performed at 20 bar pressure for 20 h, but only minor aldehyde formation (<1%) was found during analysis, probably due to oxidation of the catalyst during the recycling manipulations (entry 8). Hence, future work focusing on improving the recycling procedure is required to evaluate the potential for recyclability of the polymeric catalyst system.

## 4. Conclusions

This work extends the family of ligand-functionalized core-crosslinked micelles, previously developed in one of our laboratories, to include the nixantphos ligand. For this purpose, a nixantphos-functionalized styrene has been developed and incorporated as a co-monomer in the CCM synthesis, although the molar amount of nixantphos-styrene in the polystyrene core was limited to 1% for solubility reasons. The application of the nixantphos@CCM polymer as nanoreactor to the aqueous biphasic hydroformylation of 1-octene produced, as expected, *n*-nonanal with excellent regioselectivity, but only with a moderate activity, which may be attributed to mass transport limitation. The performance may be improved in further work by modification of the CCMs core and/or shell. The nixantphos-styrene monomer described here may also be incorporated into other kinds of polymeric or hybrid supports for catalytic applications under a wide variety of experimental conditions.

## Figures and Tables

**Table 1:** Hydroformylation of 1-octene using Rh-nixantphos@CCM catalyst ^a^.

Entry	Solvent	Rh Loading ^b^ (mol%)	P/Rh Ratio (mol/mol)	Time (h)	Pressure (bar)	Yield *n*-nonanal ^c^ (%)	Isomers ^d^
1	decanal	0.18	4	3	20	7	yes
2	heptanal	0.18	4	3	20	5	yes
3	dodecane	0.18	4	3	20	0	no
4	decanal	0.36	2	3	20	12	yes
5	dodecane	0.36	2	20	20	13	traces
6	dodecane	0.36	2	72	20	14	yes
7	dodecane	0.36	2	20	30	28	traces
8 ^e^	dodecane	0.36	2	20	20	<1	traces

^a^ Reaction conditions: 90 °C, CO/H_2_ mixture (1:1), organic phase/aqueous phase (2:1 *v*/*v*). ^b^ Rh loading relative to substrate. ^c^ Calculated by means of GC-MS, ^d^ Isomerization products of 1-octene at the studied reaction conditions; ^e^ Recycling test of entry 5.

## Supplementary Materials

The following are available online at https://www.mdpi.com/2073-4360/12/5/1107/s1, Figure S1: ^31^P{^1^H} NMR of compound **1** in CDCl_3_, Figure S2: ^1^H{^31^P} COSY NMR of compound **1** in CDCl_3_, Figure S3: ^1^H-^13^C{^31^P} HMQC of compound **1** in CDCl_3_, Figure S4: Comparison of ^13^C{^1^H} (below) and ^13^C{^1^H,^31^P} (above) NMR spectra of **1** in CDCl_3_ for selected expanded regions of the spectrum, Figure S5: ^1^H-^13^C HMBC of compound **1** in CDCl_3_, Figure S6: ^1^H NMR of compound **2** in CDCl_3_, Figure S7: ^31^P{^1^H} NMR of compound **2** in CDCl_3_, Figure S8: ^1^H NMR spectrum of nixantphos@CCM in THF-*d*_8_, Figure S9: ^1^H NMR spectrum of nixantphos@CCM in D_2_O before and after toluene swelling, Figure S10: Deconvolution of the ^1^H NMR CH_2_ proton resonance.
