# Supplementary material for: Synthesis of Nixantphos Core-Functionalized Amphiphilic Nanoreactors and Application to Rhodium-Catalyzed Aqueous Biphasic 1-Octene Hydroformylation
Source: Polymers (Basel). 2020 May 12;12(5):1107. doi: 10.3390/polym12051107 (PMC7285327; doi:10.3390/polym12051107)
Supplement: Supplementary file 1 [file polymers-12-01107-s001.pdf]

Synthesis of nixantphos core-functionalized amphiphilic nanoreactors and application to rhodium-catalyzed aqueous biphasic 1-octene hydroformylation

Ahmad Joumaa [1], Florence Gayet [1], Eduardo J. Garcia-Suarez [2], Jonas Himmelstrup [2], Anders Riisager [2], Rinaldo Poli [1,3,*] and Eric Manoury [1,*]

[1] CNRS, LCC (Laboratoire de Chimie de Coordination), Université de Toulouse, UPS, INPT, 205 route de Narbonne, BP 44099, F-31077 Toulouse Cedex 4, France CNRS
[2] Technical University of Denmark, Department of Chemistry, Centre for Catalysis and Sustainable Chemistry, Kemitorvet, Building 207, 2800 Kgs. Lyngby, Denmark
[3] Institut Universitaire de France, 1, rue Descartes, 75231 Paris Cedex 05, France
* Correspondence: rinaldo.poli@lcc-toulouse.fr, eric.manoury@lcc-toulouse.fr; Tel.: +33-561333173 (R.P.), +33-561333174 (E.M.)

SUPPORTING INFORMATION

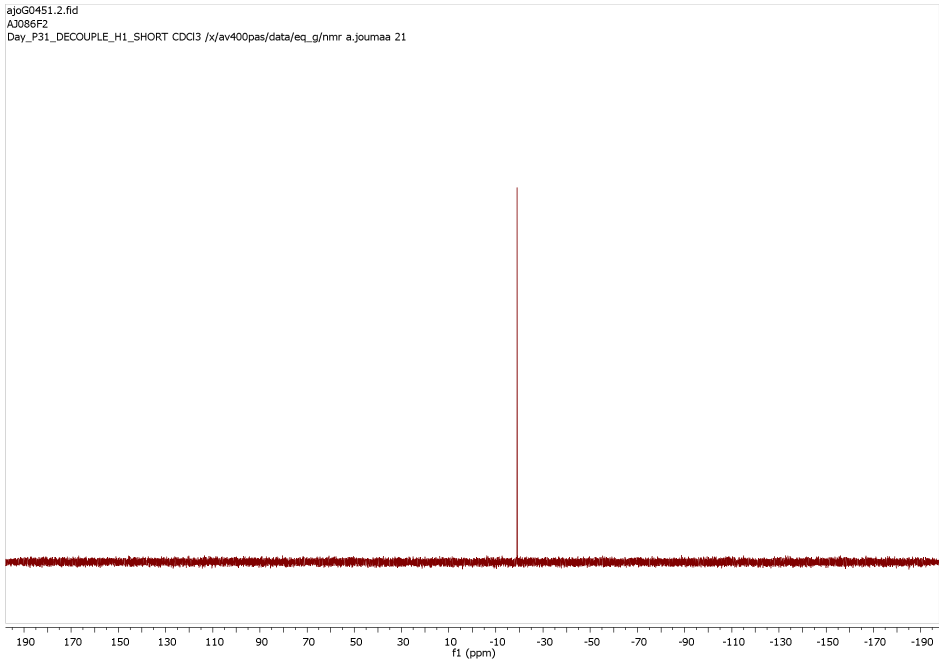

Figure S1. $^{31}P\{^{1}H\}$ NMR of compound **1** in $CDCl_3$.

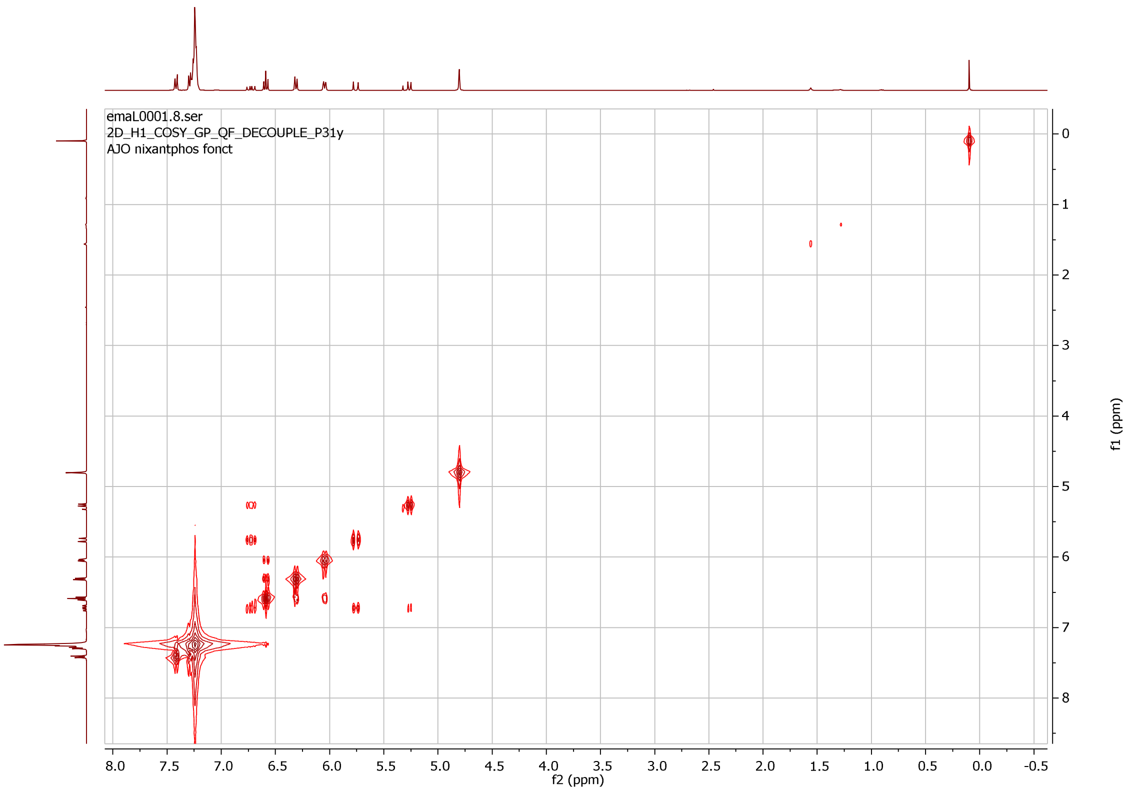

Figure S2. $^{1}H\{^{31}P\}$ COSY NMR of compound **1** in $CDCl_3$.

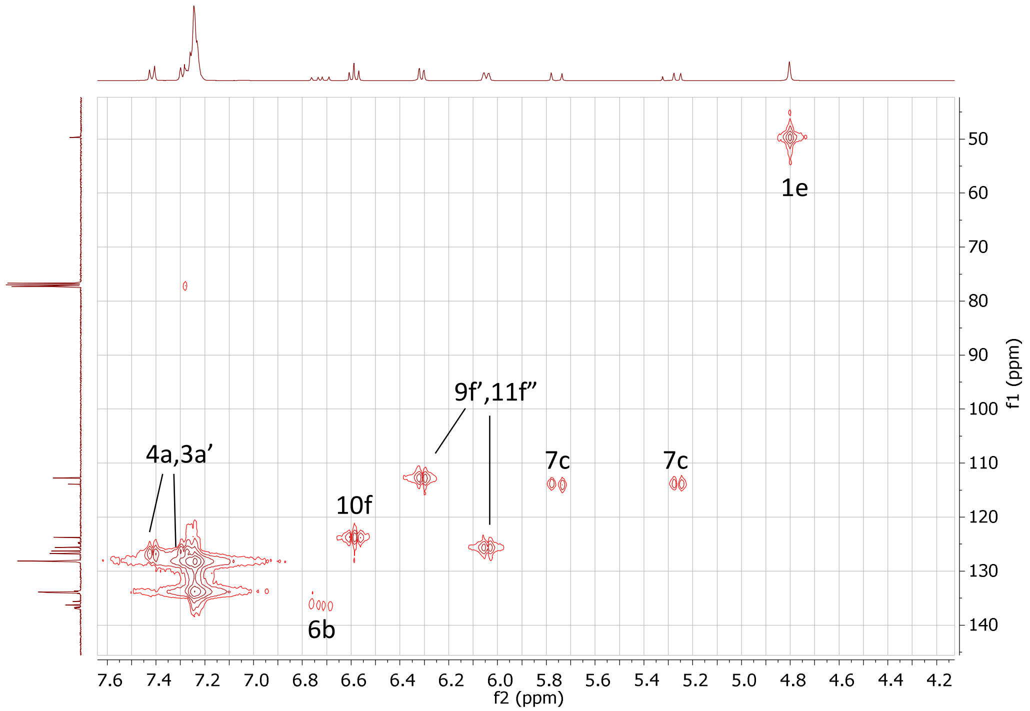

Figure S3. ^{1}H-$^{13}C\{^{31}P\}$ HMQC of compound **1** in $CDCl_3$.

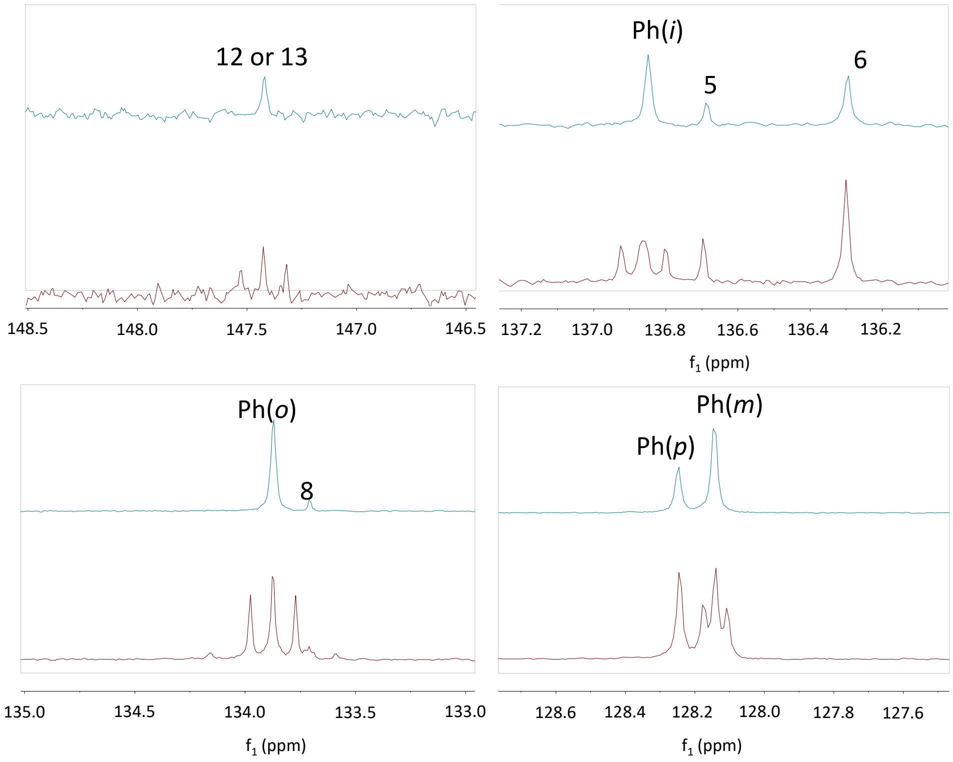

Figure S4. Comparison of $^{13}C\{^{1}H\}$ (below) and $^{13}C\{^{1}H,^{31}P\}$ (above) NMR spectra of **1** in $CDCl_3$ for selected expanded regions of the spectrum.

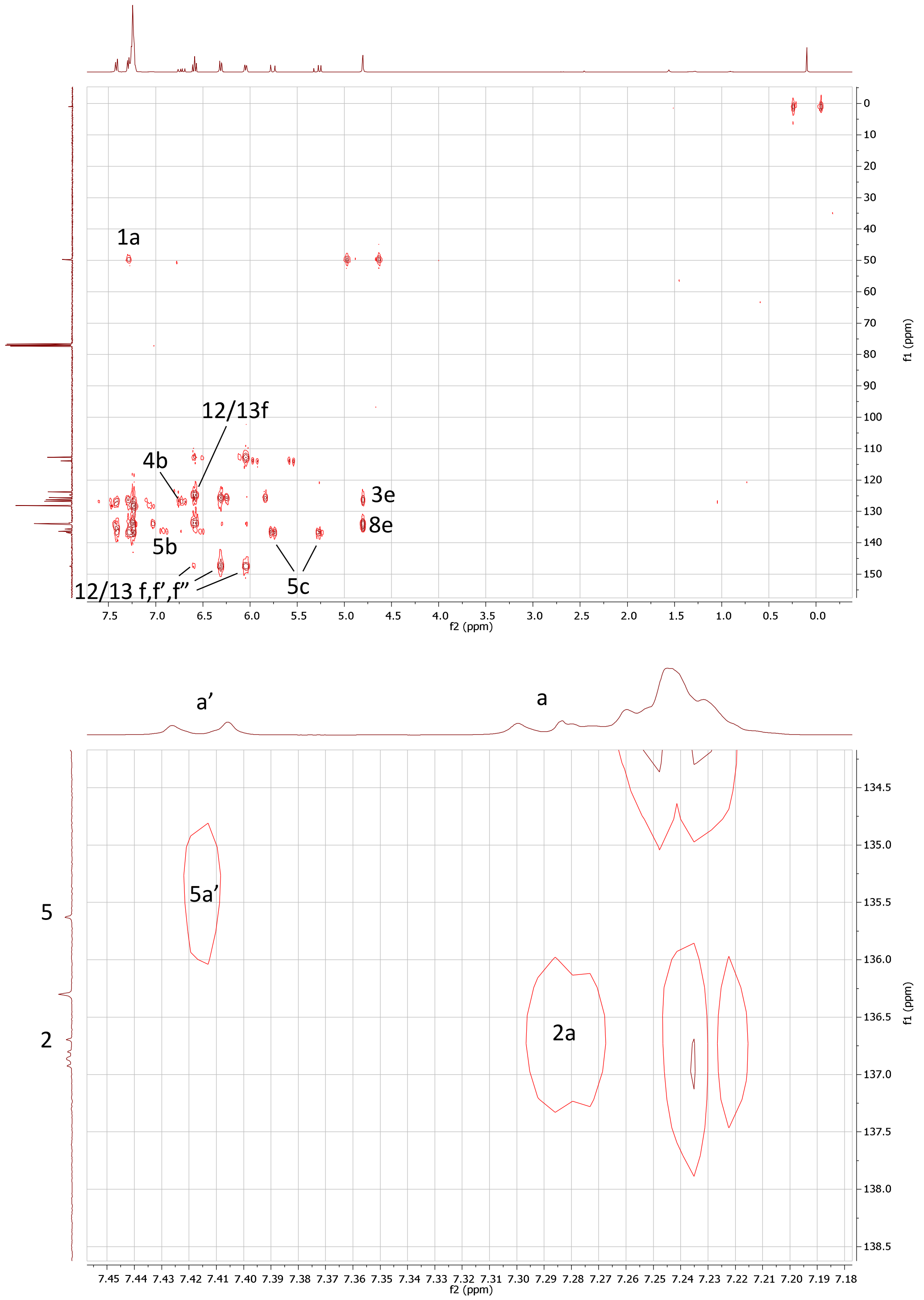

Figure S5. ^{1}H-^{13}C HMBC of compound **1** in $CDCl_3$.

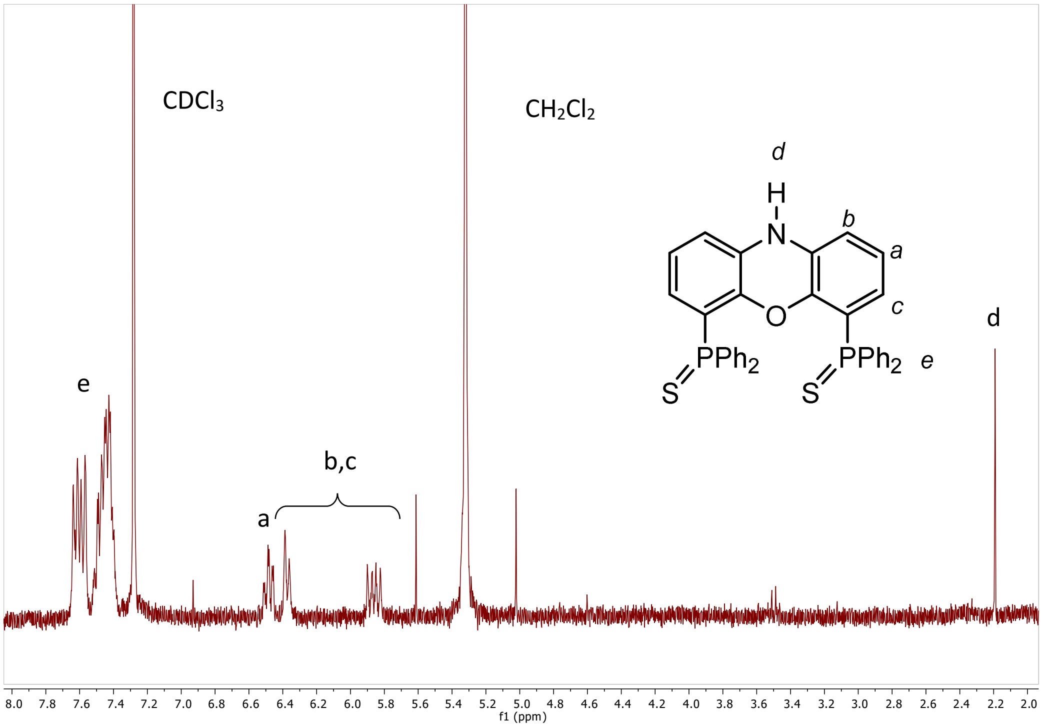

Figure S6. ^{1}H NMR of compound **2** in $CDCl_3$.

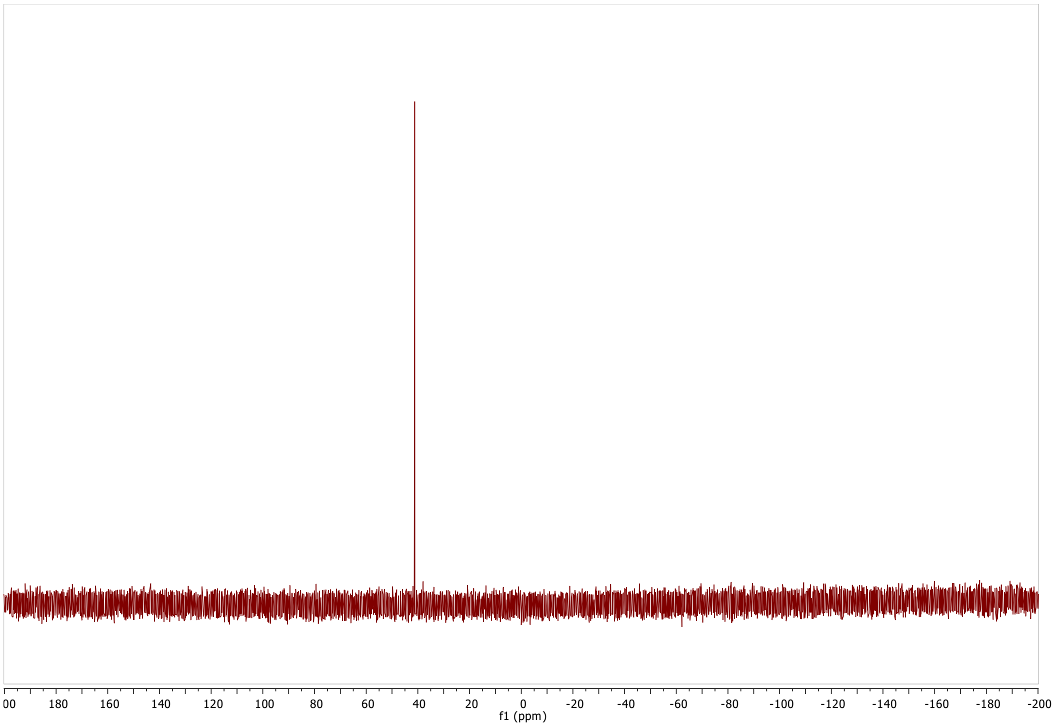

Figure S7. $^{31}P\{^{1}H\}$ NMR of compound **2** in $CDCl_3$.

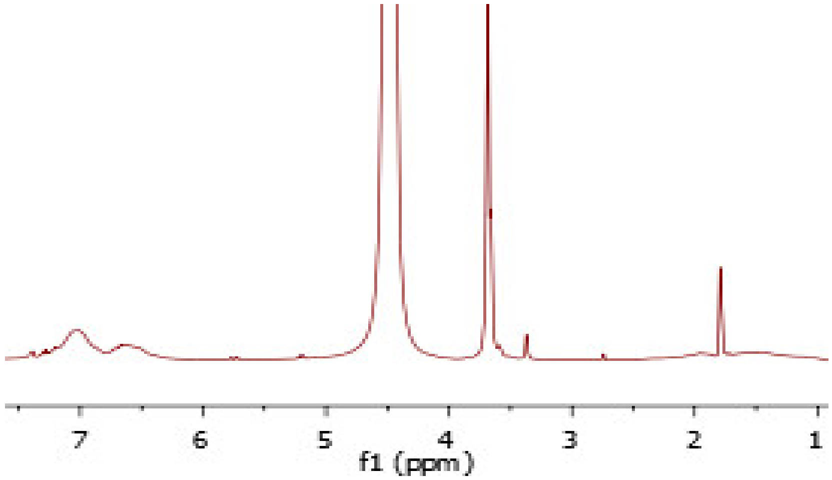

Figure S8. ^1H NMR spectrum of Nixantphos@CCM in THF-d_8.

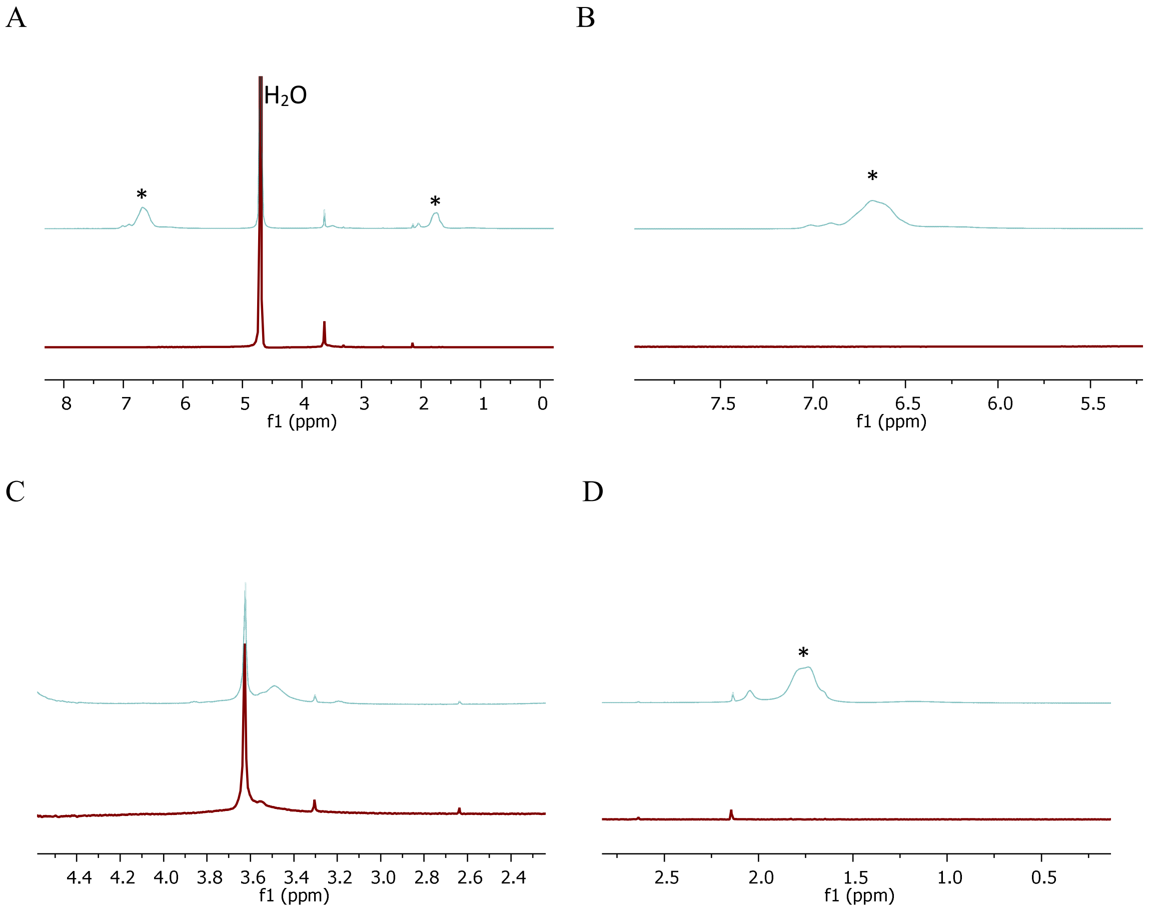

Figure S9. A: ^1H NMR spectrum of Nixantphos@CCM in D_2O before (red) and after (light blue) toluene swelling. B-D: Expansions of selected regions. The resonances marked by an asterisk belong to the swelling toluene molecules.

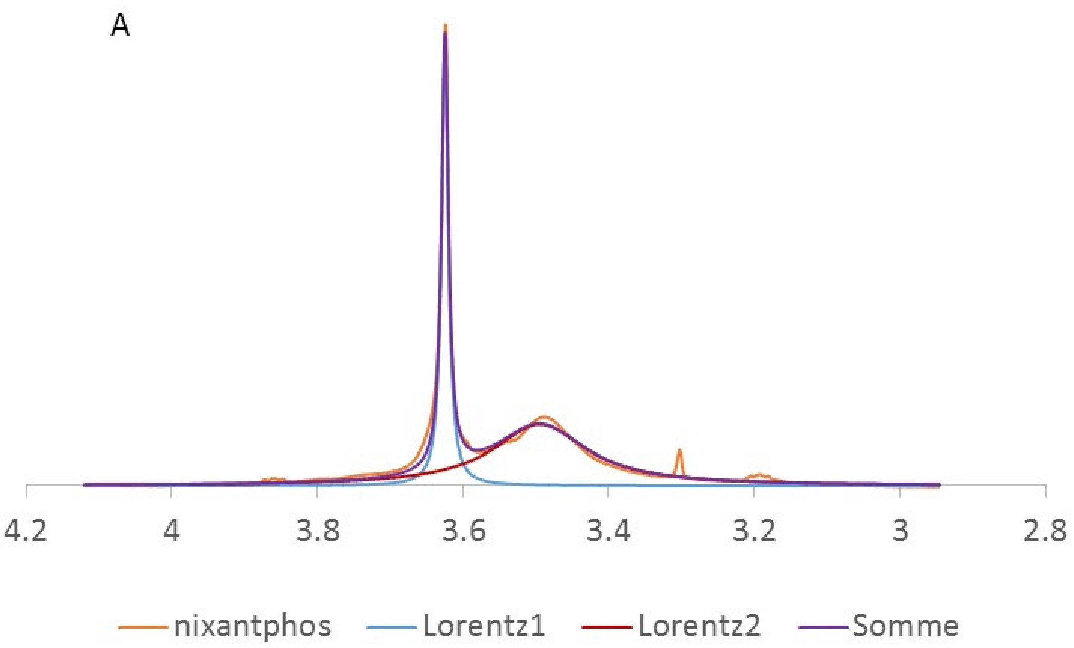

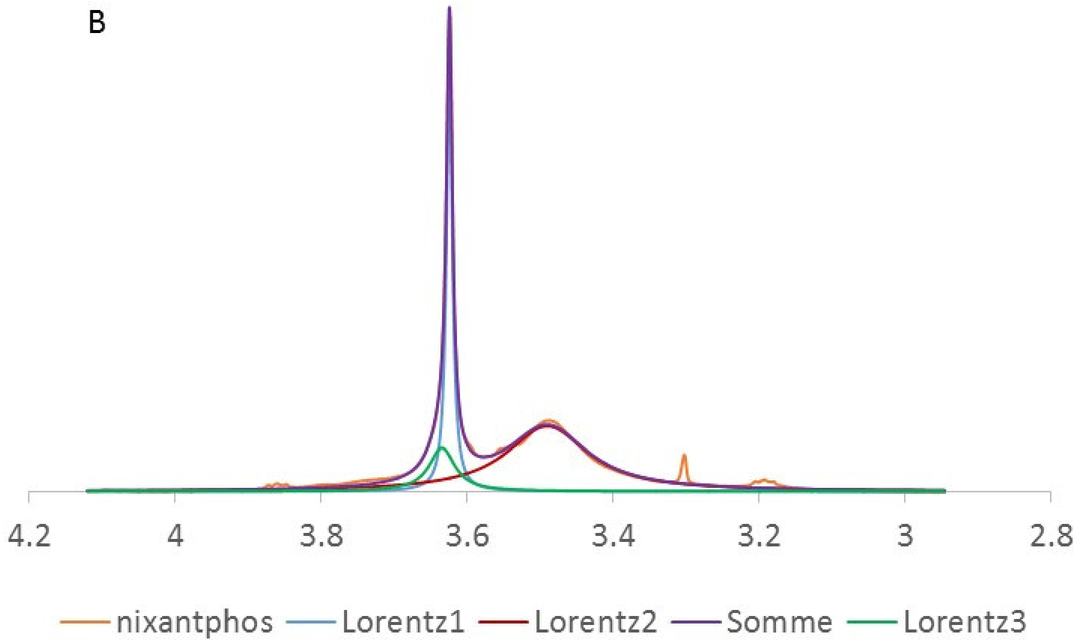

Figure S10. Expansion of the ^{1}H NMR spectrum of toluene-swollen Nixantphos@CCM in D_2O in the PEO resonance region and deconvolution of the CH_2 proton resonance as sum of two (A) or three (B) Lorentzian functions.

Interpretation of the PEO resonance region (following ref. [1])

The proton resonances of the PEO chains (CH_2 and CH_3) are split into two signals, one broader and one sharper, because of a core-shell interface structuring, placing a fraction of the chains inside the core while the others are solvated in the aqueous phase. The sharper resonances at δ 3.62 (for the CH_2 protons) and 3.30 (for the CH_3 protons at the chain end) are assigned to the more mobile chains in the water phase and the broader ones at δ 3.49 (for the CH_2 protons) and 3.19 (for the CH_3 protons) belong to the chains solvated by toluene within the core. The deconvolution of the larger CH_2 resonance as sums of Lorentzian functions provides the fraction of the chains in each phase. While the use of two Lorentzian functions was found to provide an excellent fit for the ^1H NMR spectrum of the previously reported TPP@CCM,[1] in the present case the agreement between experimental and simulated spectrum is of lower quality, see Figure S7A. A better fit can be obtained using the sum of three Lorentzian functions, as shown in Figure S7B. The optimized fractions are of 29.7 % for the sharp peak of the water-solvated protons (Lorentz 1 at δ 3.62), 57.5 % for the broader peak of the toluene-solvated protons in the core (Lorentz 2 à δ 3.49) and 12.8 % for the third population (Lorentz 3 at δ 3.64). This latter population is probably caused by a few CH_2 functions that are located near the water-toluene interphase.

[1] S. Chen, F. Gayet, E. Manoury, A. Joumaa, M. Lansalot, F. D'Agosto and R. Poli, *Chem. Eur. J.* **2016**, *22*, 6302 – 6313.
